# *Ole-e-1* Interacts with *FWL* Genes to Modulate Cell Division and Determine Fruit Size in Pears

**DOI:** 10.3390/ijms26188804

**Published:** 2025-09-10

**Authors:** Jingyi Sai, Yue Wen, Yan Zhang, Xiaoqiu Pu, Chen Chen, Lei Wang, Mengli Zhu, Jia Tian

**Affiliations:** 1 College of Horticulture, Xinjiang Agricultural University, Urumqi 830052, China; cyberone12@163.com (J.S.); wenyue900701@163.com (Y.W.); 15699377561@163.com (Y.Z.); m18434365917@163.com (C.C.); ewlei@163.com (L.W.); z1524795634@163.com (M.Z.); 2 College of Horticulture and Forestry, Tarim University, Alar 843300, China; puxiaoqiu5425@163.com

**Keywords:** pear, FWL, Ole-e-1, cell division, fruit size

## Abstract

The *fw2.2* (*fruit weight 2.2*) gene negatively regulates cell division and significantly influences fruit size, but its regulatory mechanisms in pears remain unclear. Here, we investigated how pear *FWL* (*fw2.2-like*) genes control cell division using Duli pear, Korla fragrant pear, and Yali pear. During the cell division phase, fluorescence in situ hybridization (FISH) revealed stronger expression of *FWL1* and *FWL5* in smaller fruits compared to larger ones, with both genes localized in the core and flesh tissues. Gene silencing experiments demonstrated that silencing of FWL5 leads to a significant increase in the number of cells, with a concomitant enlargement of the fruit. Yeast two-hybrid screening identified 147 proteins interacting with FWL5, showing substantial overlap with FWL1 interactors. Key candidates included metallothionein-like protein (MT) and Ole-e-1, with the latter displaying a positive correlation with fruit size during cell division. Bimolecular fluorescence complementation (BiFC) confirmed direct interactions between Ole-e-1 and both FWL1/FWL5. Functional analysis indicated the *Ole-e-1* gene family has diverse roles in pear development. We propose that Ole-e-1 interacts with *FWL* genes to modulate cell division, thereby determining final fruit size. This study uncovers a novel regulatory axis linking cell cycle control and fruit size in pears.

## 1. Introduction

Fruit size is a vital economic trait of fruit trees, a quantitative trait with inheritance influenced by both cell size and cell number. Numerous studies highlight that variation in fruit size primarily stems from differences in cell numbers [1,2,3,4]. The disparity in fruit size between cultivated varieties and wild species can be drastic, with differences sometimes reaching hundred- or thousand-fold. For instance, in tomatoes, hybridization studies that explored different small-sized wild species and large-sized cultivars have identified nearly 30 quantitative trait loci (QTLs) associated with fruit size [5,6,7]. One significant QTL, known as *fruit weight 2.2* (*fw2.2*), has been shown to account for 30% of the variation in fruit weight by negatively regulating cell division [8,9,10,11,12]. To date, over 140 homologous genes related to *fw2.2* have been isolated across various species. The *fw2.2-like genes* (*FWLs*) found in avocado, pear, and *Physalis* species act as negative regulators of fruit cell division [13,14,15]. Furthermore, in maize, genes homologous to *fw2.2* are classified as *Cell Number Regulators* (*CNRs*), with transgenic experiments demonstrating that *CNR1* can influence the overall size of the plant [16]. Subsequently, the *fw2.2* gene family has been confirmed to possess extensive regulatory functions. In rice, *OsFWL3* negatively regulates grain size, *OsFWL5* influences plant height, and *OsFWL4* is involved in the regulation of tiller number, grain length, and overall plant yield [17,18]. Additionally, *GmFWL1* plays a critical role in soybean nodule organogenesis [19], while *PavCNR12* and *PavCNR20* exhibit a significant correlation with fruit size in cherry [14]. In apples, functional analysis has shown that *MdCNR8* may control fruit size and root growth [20].

FW2.2 has been recognized as a membrane protein [21]. Regarding its mechanism of fruit size regulation, two primary perspectives have emerged. The first perspective, derived from studies on tomato, suggests that *fw2.2* interacts with casein kinase II (CKII), participating in cell cycle signaling and regulation, thereby influencing fruit size [21]. The second perspective, derived from studies on *Physalis*, proposes that *PfCNR1* (also known as *Physalis Organ Size 2*, *POS2*) interacts with the MADS-box transcription factor *PfAG2* (*AGAMOUS*-*like*), transmitting regulatory signals through specific chemical modifications. This interaction enables the transcription factor to enter the cell nucleus, bind to the promoter of PfCyclinD2;1, and inhibit its expression. Consequently, cell cycle progression is impaired due to the downregulation of PfCyclinD2;1, affecting overall cell division dynamics [14]. Similar pathways have also been observed in loquat research [22]. Recent studies have suggested that *fw2.2* has a regulatory role in cell-to-cell communication by modulating plasmodesmata (PD) transport capacity and trafficking of signaling molecules during fruit development [23,24]. Despite extensive studies, *fw2.2*’s precise biological roles and mechanisms in regulating cell division in pears remain poorly understood.

The Korla fragrant pear (*Pyrus* × *sinkiangensis* Yü), a member of the subfamily Pomoideae within the family Rosaceae, is distinguished by its thin skin, crisp flesh, unique aroma, and good storability [25]. The economic value of Korla fragrant pears is significantly influenced by fruit size, with fruits weighing 120 g commanding prices 2 to 3 times higher than those under 80 g. However, in practical cultivation, the average weight of individual Korla fragrant pears typically does not exceed 100 g, leading to a reduction in edible portions and subsequently impacting the economic viability and progression of the Korla fragrant pear industry. Previously, we isolated homologous genes, *FWL1* and *FWL5*, of *fw2.2* from pears, identifying an inverse relationship between their expression levels and fruit numbers during cell division. This suggests a significant association between these genes and fruit size in pears [15,26]. As membrane proteins, FWL1 and FWL5 are indirectly involved in these functional roles. In our research, we identified two potential interacting proteins, MT (metallothionein-like protein) and EGF (collagen- and calcium-binding EGF domain-containing protein 1), which may play a role in cell division, through the construction of a yeast two-hybrid expression library for FWL1 [27]. To elucidate the pathways by which pear *FWL* genes regulate fruit size, we selected Duli pear (*Pyrus betulifolia* Bunge), Korla fragrant pear, and Yali pear (*Pyrus bretschneideri*) for analysis. Using fluorescence in situ hybridization (FISH), we investigated the specific expression sites of *FWL1* and *FWL5*. In addition, the VIGS vectors for FWL1 and FWL5 were constructed, which were then used to infect young fruits, followed by phenotypic observation. We also constructed a yeast two-hybrid expression library for *FWL5* and analyzed the expression levels of MT and EGF during critical cell division stages, integrating the results from our prior *FWL1* screening. Our findings indicate that *Ole-e-1* interacts with both *FWL1* and *FWL5*, exhibiting a positive correlation with fruit number and suggesting its potential role in influencing pear fruit size.

## 2. Results

### 2.1. Tissue Fluorescence In Situ Hybridization Analysis of FWL1 and FWL5

**Figure 1:**
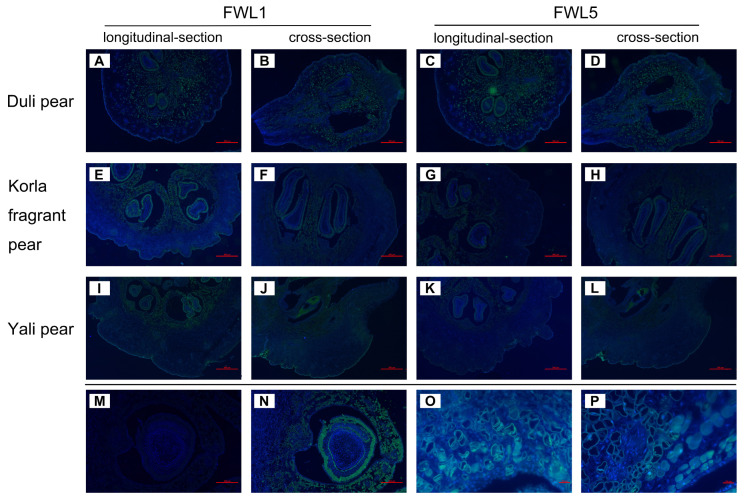
Results of in situ hybridization of FWLs in pear pulp at 10 DAFB. The notations (**A**–**D**) correspond to the Duli pear, (**E**–**H**) correspond to the Korla fragrant pear, and (**I**–**L**) correspond to the Yali pear. Specifically, (**A**,**E**,**I**) represent the hybridization results of the FWL1 antisense probe applied to the longitudinal sections of the respective fruits. In contrast, (**B**,**F**,**J**) illustrate the hybridization results of the FWL1 antisense probe on the cross-sections of the same fruits. Additionally, (**C**,**G**,**K**) reflect the hybridization results of the FWL5 antisense probe on the longitudinal sections for Duli pear, Korla fragrant pear, and Yali pear, respectively. (**D**,**H**,**L**) showcase the hybridization results of the FWL5 antisense probe on the cross-sections of these fruits. (**M**) denotes the hybridization results of the sense probe, while (**N**) represents the results of the antisense probe. Lastly, (**O**,**P**) depict the hybridization results of the antisense probe observed under a 40× microscope. All samples are at 10 DAFB. The scale bars for (**A**–**N**) represent 500 µm, while for (**O**,**P**), the scale bars denote 100 µm.

To elucidate the role of *FWL1* and *FWL5* genes in pear fruit development, we analyzed their spatial expression patterns during the critical phase of cell division (10 DAFB) in the fruits of Duli pear, Korla fragrant pear, and Yali pear. Utilizing FISH, our findings revealed that the hybridization signals for both *FWL1* and *FWL5* were predominantly localized in the core of the pear flesh tissues, exhibiting a gradient decrease outward until the signals were no longer detectable. Cross-section and longitudinal assessments consistently demonstrated that the intensity of these hybridization signals was the highest in Duli pear, followed by Korla fragrant pear, with the lowest in Yali pear. This observation aligns with previously acquired fluorescence quantitative data, reinforcing the notion that *FWL1* and *FWL5* display higher signal intensity in smaller fruits compared to larger cultivars, suggesting a potentially broader functional range in smaller fruit phenotypes. Notably, no signal was detected in the hybridization involving the Justice Probe. In contrast, the antisense probe hybridization indicated the presence of signals within the cytoplasm, particularly localized around the cell membrane. These results further support the conclusion that *FWL1* and *FWL5* are likely membrane-associated proteins (Figure 1).

### 2.2. Silencing of FWL Genes via TRV-Based VIGS

**Figure 2:**
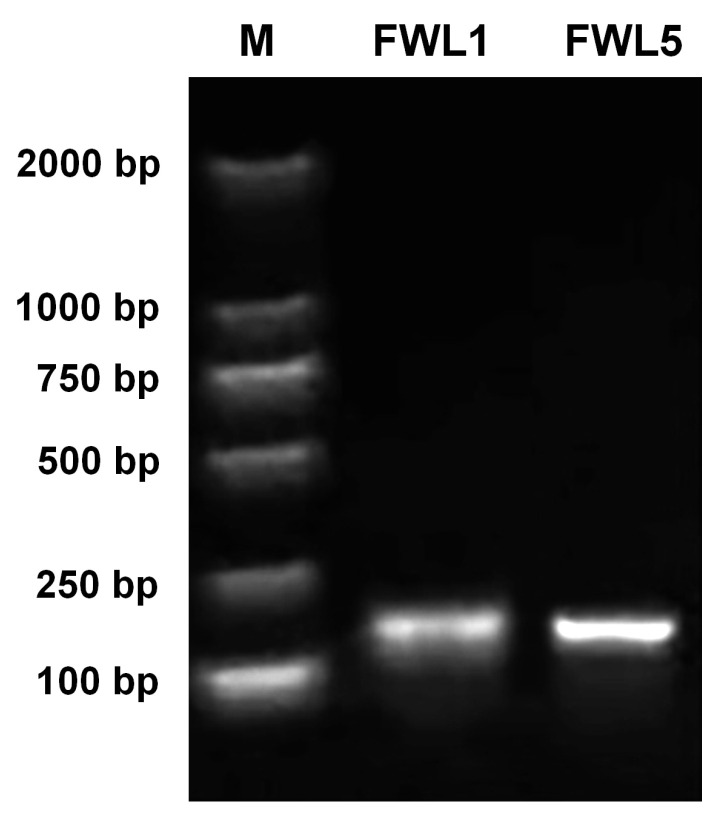
PCR amplification of *FWL1* and *FWL5* fragments for TRV-VIGS vector construction.

To further investigate the functions of *FWL1* and *FWL5*, virus-induced gene silencing (VIGS) experiments were performed. Total RNA was extracted from Korla fragrant pear fruits at the critical stage of cell division, and cDNA was synthesized via reverse transcription. Specific primers for *FWL1* and *FWL5* were used for PCR amplification, yielding target fragments of 133 bp and 128 bp in length, respectively. After verification by sequencing, the fragments were recovered, ligated, and transformed, resulting in the successful construction of the recombinant expression vectors pTRV2-*FWL1* and pTRV2-*FWL5* (Figure 2).

**Figure 3:**
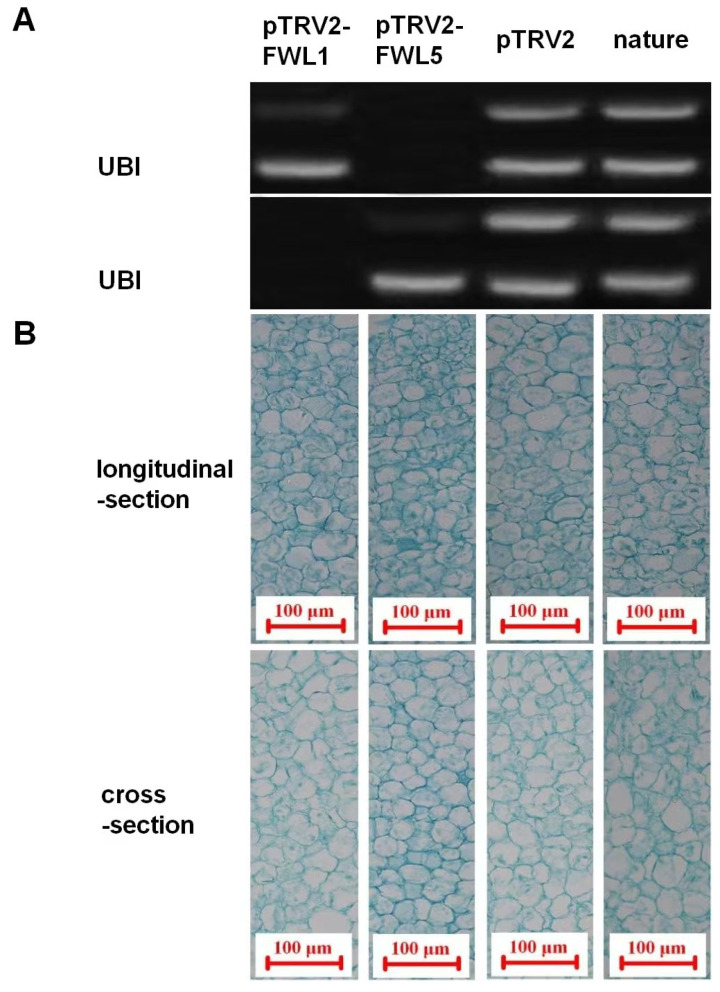
Effects of pear fruit on *FWL1/5* silencing at 10 days post-injection. (**A**): Expression levels of *FWL1/5*. (**B**): Paraffin sections of fruit tissues.

The injection method was employed to infect young fruits at 10 DAFB, with fruits injected with the empty vector (pTRV2) and naturally growing fruits set as controls. Ten days after infection, pulp tissues from each treatment group were collected for semi-quantitative RT-PCR analysis. The results showed that the expression levels of *FWL1* and *FWL5* genes were inhibited in young fruits of the silenced groups, while normal expression was maintained in both control groups, indicating that *FWL1* and *FWL5* genes were successfully silenced in young Korla fragrant pear fruits (Figure 3).

### 2.3. Effects of FWL Gene Silencing on Fruit Size, Cell Size, and Cell Number

The weight and volume of young fruits in each group were measured (Table 1). The results demonstrated that young fruits with FWL5 silencing exhibited the largest weight and volume, which were significantly higher than those in the empty vector control, natural growth control, and FWL1-silenced groups. To further explore the cytological basis, paraffin sections of pulp tissues were prepared, and cell volumes were measured. It was found that there was no significant difference in cell size between the FWL1-silenced group and the two control groups, but significant differences were observed between these groups and the FWL5-silenced group. Statistical analysis of cell numbers showed that the FWL5-silenced group had the highest number of cells, significantly higher than all other treatment groups. These results suggest that silencing FWL5 can affect fruit development by promoting the division of pulp cells (increasing cell numbers) in Korla fragrant pears.

### 2.4. Library Screening and Identification of Candidate Interacting Proteins

**Figure 4:**
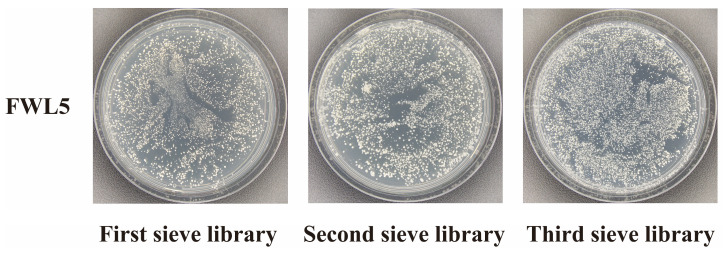
Sieve library conversion efficiency.

We conducted a comprehensive screening across three distinct libraries for FWL5. The statistical analysis of transformation efficiency indicated that each screening yielded over 1000 plaques and a transformation rate of at least 1.0 × 10^6^ CFU, meeting the requisite criteria for successful library screenings (Figure 4). Upon evaluating the positive clones, we identified that the FWL5 screening yielded more than 150 plaques. Subsequently, the positive clones were sequenced, resulting in a total of 143 successfully sequenced clones. The screened proteins underwent a BLAST comparison within the NCBI database. The analysis revealed the presence of 25 unknown proteins among the interacting proteins. Notably, the proteins exhibiting the highest frequency of interactions were metallothionein-like protein type 2, metallothionein-like protein (Met1), and the Pyrus bretschneideri branchpoint-bridging protein-like (Table 2).

### 2.5. Self-Activation and Functional Assay of Decoy Expression Vectors

**Figure 5:**
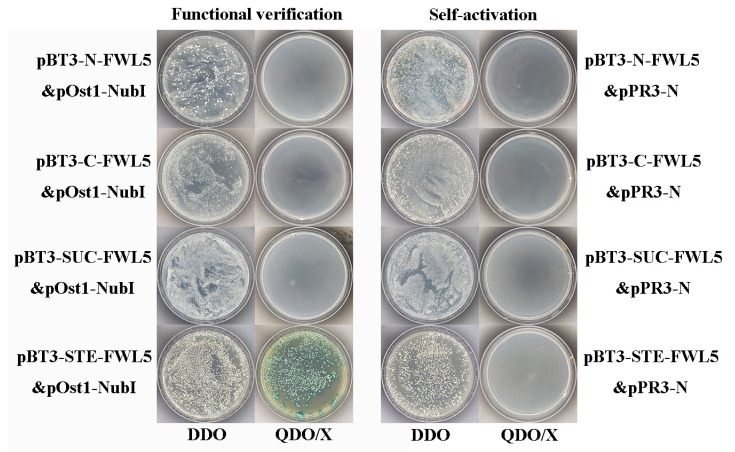
Decoy expression vector function and self-activation detection.

To assess the functionality of the bait protein in yeast, four distinct functional verification combinations were co-transformed into the NMY51 strain of yeast. The results indicated that combinations pBT3-N-FWL5&pOst1-NubI, pBT3-C-FWL5&pOst1-NubI, and pBT3-SUC-FWL5&pOst1-NubI exhibited normal growth on DDO (SD-Leu-Trp) medium; however, they failed to grow on QDO/X (SD/-Leu/-Trp/-His/-Ade/X-a-ga) medium. In contrast, the combination pBT3-STE-FWL5&pOst1-NubI not only grew on DDO (SD-Leu-Trp) medium but also demonstrated growth and blue coloration on QDO/X (SD/-Leu/-Trp/-His/-Ade/X-a-ga) medium. This observation indicates that pBT3-STE-FWL5 effectively expresses the fusion protein in yeast and activates the downstream reporter gene. All four combinations used for auto-activation detection grew successfully on the DDO (SD-Leu-Trp) medium, confirming the success of the co-transformation process. However, their limited growth on QDO/X (SD/-Leu/-Trp/-His/-Ade/X-a-ga) selective medium suggests a low level of auto-activation activity and minimal interaction between the bait protein and the empty library plasmid (Figure 5).

### 2.6. Expression Analysis of Candidate Interactions Protein-Related Genes in Pear Fruit During Critical Cell Division Stages

**Figure 6:**
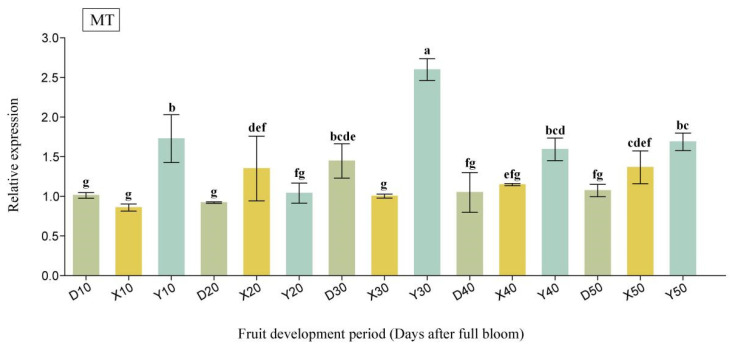
The qRT-PCR analysis of MT at the critical stage of cell division in pear fruit. The horizontal axis represents different pear cultivars and DAFB. “D” stands for Duli pear, “X” for Korla fragrant pear, and “Y” for Yali pear. The numbers 10–50 indicate 10–50 DAFB. Different lowercase letters (a, b, c, d, e, f, g) indicate significant differences at *p* < 0.05.

**Figure 7:**
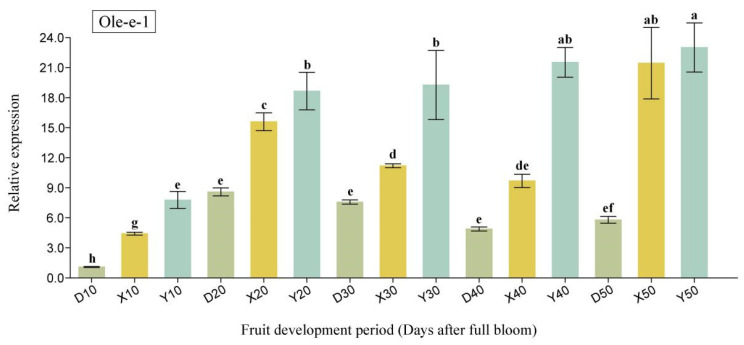
The qRT-PCR analysis of Ole-e-1 at the critical stage of cell division in pear fruit. The horizontal axis represents different pear cultivars and DAFB. “D” stands for Duli pear, “X” for Korla fragrant pear, and “Y” for Yali pear. The numbers 10–50 indicate 10–50 DAFB. Different lowercase letters (a, b, c, d, e, f, g, h) indicate significant differences at *p* < 0.05.

In this study, we identified two candidate proteins, MT and Ole-e-1, based on their occurrence times and associated functions. The qRT-PCR analyses revealed that the expression level of the MT gene was consistently highest in Yali pear throughout the critical cell division period (10 DAFB to 50 DAFB), except for a notable decline at 20 DAFB. Specifically, between 10 DAFB and 30 DAFB, the expression hierarchy observed was Yali pear > Duli pear > Korla fragrant pear. Conversely, during the 40 DAFB-50 DAFB window, larger fruit types demonstrated greater expression levels compared to smaller fruit types, with the following ranking: Yali pear > Korla fragrant pear > Duli pear. Notably, no specific expression pattern emerged across the entire time frame analyzed (Figure 6). The *Ole-e-1* gene exhibited a consistent expression trend throughout the cell division period, with relative levels measured as Yali Pear > Korla fragrant pear > Duli pear. This gene displayed high expression in larger fruit varieties and lower expression in smaller ones, indicating a positive correlation with fruit size (Figure 7).

### 2.7. Validation of FWL1/5 Interactions with Ole-e-1

**Figure 8:**
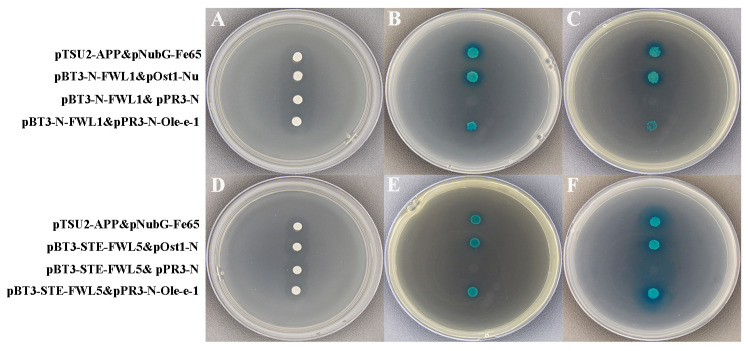
Rotary validation of FWL1/5 with Ole-e-1. (**A**) SD/-Trp/-Leu medium; (**B**) SD/-Trp/-Leu/-His/X-a-gal medium; (**C**) SD/-Trp/-Leu/-His/-Ade/X-a-gal medium; (**D**) SD/-Trp/-Leu medium; (**E**) SD/-Trp/-Leu/-His/X-a-gal medium and (**F**) SD/-Trp/-Leu/-His/-Ade/X-a-gal medium.

The pBT3-N-FWL vector was generated through homologous recombination. Subsequently, the pBT3-N-FWL and pPR3-N-Ole-e-1 constructs were co-transformed into yeast receptor cells for validation of interactions. In contrast, the negative controls (pBT3-N-FWL1 with pPR3-N and pBT3-STE-FWL5 with pPR3-N), tested on SD/-Trp/-Leu/-His/X-a-gal/50mM3-AT and SD/-Trp/-Leu/-His/-Ade/X-a-gal/50mM3-AT medium, exhibited no growth. Conversely, the positive controls, including pTSU2-APP&pNubG-Fe65, pBT3-N-FWL1&pOst1-NubI, pBT3-N-FWL1&pPR3-N-Ole-e-1, pBT3-STE-FWL5&pOst1-NubI, and pBT3-STE-FWL5&pPR3-N-Ole-e-1, demonstrated normal growth and turned blue. These observations indicate a potential interaction between both FWL1 and FWL5 and the Ole-e-1 proteins (Figure 8).

### 2.8. BiFC Demonstrates That FWL1/5 Interacts with Ole-e-1 Protein

**Figure 9:**
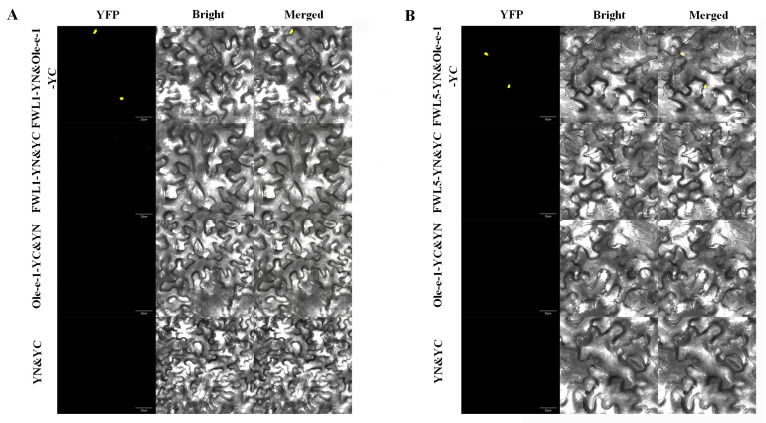
BIFC showed that FWL1 and FWL5 interacted with Ole_e_1 protein. (**A**) BIFC showed that FWL1 interacted with Ole_e_1 protein and (**B**) BIFC showed that FWL5 interacted with Ole_e_1 protein. YFP, yellow fluorescence channel; Bright, bright field; Merged, merged channel. The scale bar is 25 μm.

The BiFC assay was conducted to further elucidate the interactions between the FWL1, FWL5, and Ole-e-1 in plant systems. Observations indicated the presence of yellow fluorescent signals in tobacco leaves that had been infiltrated with the positive control combinations: pEarleyGate201-YN-FWL1&pEarleyGate202-YC-Ole-e-1 and pEarleyGate201-YN-FWL5&pEarleyGate202-YC-Ole-e-1. In contrast, the negative control combinations—pEarleyGate201-YN-FWL1&pEarleyGate202-YC, pEarleyGate201-YN-FWL5&pEarleyGate202-YC, pEarleyGate201-YN&pEarleyGate202-YC-Ole-e-1, and pEarleyGate201-YN&pEarleyGate202-YC—did not yield any yellow fluorescent signals. These findings suggest a specific interaction between Ole-e-1 and both FWL1 and FWL5 proteins within plant cells (Figure 9).

### 2.9. Ole-e-1 Bioinformatics Analysis

**Figure 10:**
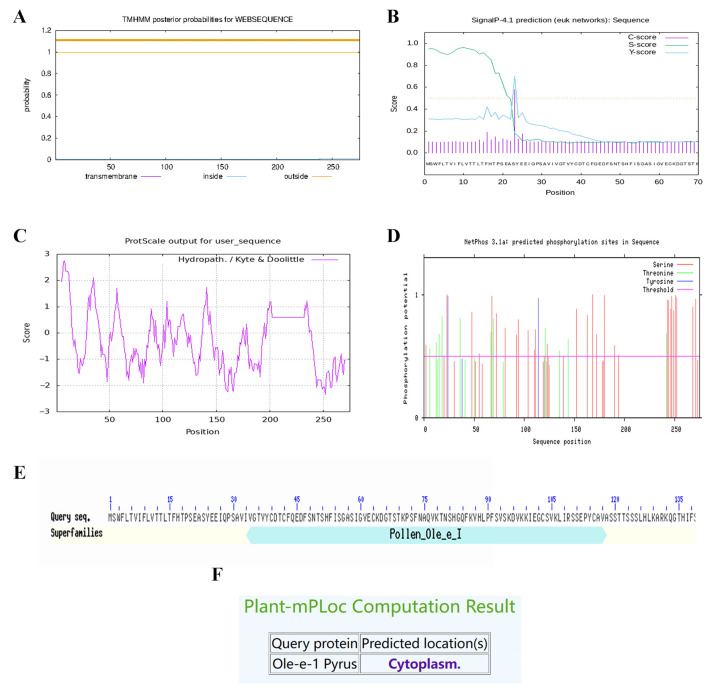
Bioinformatics analysis of Ole-e-1 protein. (**A**) Prediction of the transmembrane structure of Ole-e-1 protein. (**B**) Ole-e-1 protein signaling peptide prediction. (**C**) Hydrophobicity analysis of Ole-e-1 protein; a negative number indicates hydrophilicity, and a positive number indicates hydrophobicity. (**D**) Predictive analysis of Ole-e-1 protein phosphorylation sites. (**E**) Conserved structural domains of the Ole-e-1 protein and (**F**) Prediction of subcellular localization of Ole-e-1 protein.

The pear Ole-e-1 protein is characterized as an unstable hydrophilic protein, exhibiting a molecular weight of 28,997.97 Da and an isoelectric point of 8.83. It features a signal peptide spanning amino acids 1 to 22, lacks any transmembrane structure, and incorporates a Pollen_Ole_e_1 structural domain. Furthermore, this protein possesses 42 phosphorylation sites distributed among serine, threonine, and tyrosine residues (Figure 10).

**Figure 11:**
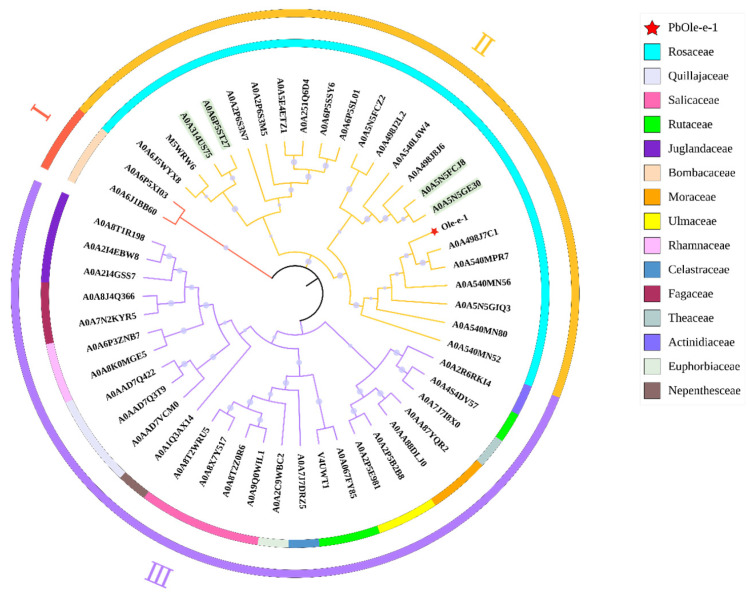
The phylogenetic tree for the Pyrus Ole-e-1 protein in relation to other plant Ole-e-1 proteins. The outer ring of the diagram illustrates the various subfamilies, designated as I, II, and III, while the inner ring delineates the specific family to which each protein is affiliated.

The Ole-e-1 protein sequence served as the target for BLAST alignment conducted using the UniProt database. Based on this analysis, the top 50 proteins were selected for phylogenetic tree construction and analysis (Figure 11). The resulting phylogenetic tree was categorized into three distinct subfamilies, encompassing species from a total of 15 families. In Subfamily I, two proteins were identified, both from the Malvaceae family. Subfamily II comprised 23 proteins exclusively from the Rosaceae family, while Subfamily III included 26 proteins representing 13 different families. Notably, the target protein Pbformin-like exhibited a close relationship with A0A498J7C1 and A0A540MPR7. Additionally, the alignment of proteins A0A5N5FCJ8, A0A5N5GE30, A0A6P5ST27, and A0A314US75 in Subfamily II revealed them to be type II Formin proteins, which have been implicated in the regulation of plant cell division. In Subfamily III, the alignment result for A0A6P5XI03, identified as a *Cyclin-dependent kinase* (*CDK*), is associated with key processes such as cell growth, proliferation, and differentiation.

## 3. Discussion

*Fw2.2* is recognized as a pivotal quantitative trait gene influencing fruit size by negatively regulating cell division [8]. Research on this gene has revealed differing mechanisms of action in tomato and *Physalis*, though both species modulate fruit size through interactions with various proteins. In pear, an early-stage analysis of *FWL1* and *FWL5* demonstrated that, similar to other members of the *Fw2.2* gene family, their expression levels were elevated in smaller fruits but diminished in larger fruits during cell division, exhibiting a negative correlation with fruit size [15,26]. To elucidate the spatiotemporal expression patterns of these two genes, FISH analysis indicated that the hybridization signals for both *FWL1* and *FWL5* gradually diminished and ultimately disappeared from the fruit core along the equatorial axis. In *Physalis*, in situ tissue hybridization revealed concentrated signals for *POS2* near the ovule and its connected placenta, implying *POS2*’s role in regulating the development of these structures [14]. In addition, our findings further indicated that the hybrid signals of *FWL1* and *FWL5* were significantly stronger in smaller fruits compared to larger ones, reinforcing the hypothesis that these genes may negatively influence fruit size development.

In prior research, the overexpression of the pear *FWL1* gene in *Arabidopsis thaliana* was observed to significantly influence organ size. In our experiment, although silencing of the *FWL1* gene was successfully achieved 10 days post-infiltration, no corresponding data indicated a discernible effect on cell division. In stark contrast, the silencing of *FWL5* was associated with an increase in cell number, ultimately leading to larger fruit sizes. In the context of maize studies, *CNR1* and *CNR2* are posited to have a co-regulatory role in cell number modulation. This hypothesis arises from the relatively low expression levels of *CNR1* in various maize tissues and organs, which render it inadequate as a standalone regulator of cell division. Nonetheless, *CNR1* displays a pronounced capability to diminish the size of maize tissues and organs in transgenic plants. Conversely, *CNR2*’s expression levels exhibit a strong negative correlation with cell division; however, transgenic plants overexpressing *CNR2* did not present any dwarfing phenotype. Thus, the potential co-regulatory relationship between *FWL1* and *FWL5* in pear necessitates further investigation.

To deepen the understanding of the mechanisms at play for *FWLs*, we constructed a three-frame cDNA yeast two-hybrid library based on the young pear fruit membrane system. The results from screening revealed consistent outcomes, with the most frequently identified proteins being MT and EGF. Previous studies have demonstrated that *OsMT2b* regulates seed germination and root development by modulating the internal levels of cytokinins in rice [28]. In addition, the functions of PvMT1A in the root development of *Phaseolus vulgaris* are achieved through the regulation of the ROS balance, which requires fine-tuning the regulation of O_2_^−^ and H_2_O_2_ levels for cell division and differentiation, respectively [29]. Among the extensive *FWL gene* family, membrane proteins associated with cadmium transport and calcium ion channels have been characterized as participants in metal ion transport across the plasma membrane, leading some scholars to suggest that the transport of divalent cations such as cadmium, calcium, or zinc might triggers signaling pathways that affect cell proliferation, thereby regulating fruit size [23,30,31]. Interestingly, the metals that are predominantly associated with the *MT* gene also include cadmium, copper, and zinc [29,32]. However, our study found no discernible expression pattern of the *MT* gene during the critical phase of cell division. This observation leads us to speculate that the *MT* gene may not play a role in regulating pear fruit size.

Moreover, EGF, recently updated in the NCBI database (June 2022) as *Pyrus bretschneideri* branchpoint-bridging protein-like (BBP) (LOC103966784), has been identified as the same protein linked to FWL5 in previous research. Historically, BBP has been recognized for its role in facilitating the recognition of introns, a function that does not strongly correlate with fruit size [33]. Nonetheless, given its Pollen_Ole_e_1 domain, we propose the nomenclature Ole-e-1. Notably, our investigation showed that Ole-e-1 exhibited a positive correlation with pear fruit size throughout the fruit cell division phase. Further validation through yeast two-hybrid and BiFC validation assays confirmed the interaction between *Ole-e-1* and two *FWL* genes.

Further analysis of the *Ole-e-1* gene family has revealed that this protein is categorized into at least 109 distinct subfamilies [34]. These subfamilies comprise extensin proteins, proline-rich proteins, hydroxyproline-rich glycoproteins, and tyrosine-rich/hydroxyproline-rich glycoproteins, among others [35]. The *Ole-e-1* gene was initially identified in olive pollen and is homologous to the tomato *Lat 52* gene [36]. Both genes are implicated in the regulation of pollen germination and pollen tube elongation, and they serve as significant allergens for humans [37,38,39]. Additional research indicates that the *Ole-e-1* gene family fulfills various roles during the growth and development of different plant species. For instance, Bruex discovered that proteins containing the Pollen_Ole_e_1 domain are involved in root hair formation. In rice, this gene exhibits tissue-specific expression during transcription, suggesting a diversity of protein functions [40]. Studies on Ole-e-1 proteins in *Arabidopsis* also indicate their potential involvement in multiple aspects of plant development [41]. In soybeans, factors such as drought, low temperatures, salt stress, and abscisic acid can induce the expression of the wild-type soybean *GmPOI* gene, signifying its association with stress resistance [42]. Furthermore, our phylogenetic analysis identified durians in Subfamily III as *CDK* genes, known for mediating the phosphorylation of various substrates and serving as core regulatory factors in the plant cell cycle [43]. These genes have been confirmed in species such as tobacco, tomato, and rice, validating their role in cell division [44,45]. Notably, *CDKs* share approximately 33% identity with *CKII*, which has been associated with cell division in tomatoes. Additionally, Formin-2-like proteins from the *Pyrus* and *Prunus* genera in Subfamily I have been shown to influence cell division in *Arabidopsis thaliana* and *Oryza sativa* [46,47,48]. Therefore, the *Ole-e-1* gene family exhibits a diverse range of functions. This study confirms that the *Ole-e-1* gene exhibits an interaction with two *FWL* genes in pear trees, while subsequent qRT-PCR experiments demonstrate that the *Ole-e-1* gene exerts a positive regulatory effect on cell division. Based on the above research, we propose a novel pathway for *FWLs* to regulate fruit size in pears: *FWL1/5* interacts with *Ole-e-1* to regulate cell division, thereby affecting fruit size. Previous studies have demonstrated that *CKII* can participate in the regulation of fruit size in tomato through its interaction with the FW2.2 protein, and this interaction pathway is also one of the key pathways for crop yield regulation [14]. In this study, the results of phylogenetic tree analysis showed that the Ole-e-1 protein shares a certain degree of homology with *CDKs*. As core regulatory factors of the cell cycle, *CDKs*, like *CKII*, have been confirmed to affect plant growth and development by regulating the cell division process and play an important role in crop yield formation [44,45]. Therefore, the *Ole-e-1* gene may possess broader biological functions during plant development and is expected to serve as a novel candidate target for the genetic improvement of fruit size.

## 4. Materials and Methods

### 4.1. Test Materials and Growth Conditions

Duli pear, Korla fragrant pear, and Yali pear were sourced from the Korla fragrant pear research center’s scientific research and experimental base located in Xinjiang (41°36′53.86″ N, 86°00′39.09″ E). This region is characterized by a temperate continental climate with four distinct seasons. Each selected variety consisted of 10-year-old trees with moderate vigor, optimal growth conditions, and consistent management. During the critical phase of pulp cell division, which occurs from 10 DAFB (days after full blooms) to 50 DAFB, thirty fruits from each variety were harvested during each period and then peeled, with their pulp excised. The pulp samples were then promptly placed in liquid nitrogen and stored at −80 °C for future analysis. The infection experiment was conducted at 10 DAFB. Fruits of the Korla fragrant pear were randomly selected from the middle part of the outer canopy for infection. At 10 days post-infection, fruit weight and longitudinal and transverse diameters were measured. The pulp tissues were then excised into small pieces and immediately preserved in liquid nitrogen and FAA fixative solution for subsequent analysis.

### 4.2. FWL1 and FWL5 Tissue Fluorescence In Situ Hybridization (FISH)

Fixed tissue samples were dehydrated at 4 °C, brought back to room temperature for transparency, infiltrated with wax, and embedded. The sample blocks were stored at 4 °C. Tissue sections were placed on a 42 °C heating stand to remove moisture, transferred to an aluminum lunch box, and then dehydrated in a 47–48 °C warm box for 36 h before storage at 4 °C. Regions with high FWL1 and FWL5 expression were selected as templates for RNA probes (sequences in Table A1; probes synthesized by Shanghai Gefan Biotechnology Co., Shanghai, China). For hybridization, after section pretreatment, sections were washed twice with 5× SSC (pH = 7.5, 1 min each), prehybridized in a wet box with prehybridization solution at 65 °C for 1 h, and then hybridized with 500 ng/mL FAM-labeled probes in the dark at 65 °C for 48 h. Post-hybridization, samples were incubated in 2× SSC (pH = 7.5) for 1 h and washed at room temperature for 1 min, followed by three washes with a 1:1 formamide/4× SSC (pH = 4.5) mixture at 60 °C or 65 °C for 20 min each and five PBS washes (1 min each). Nuclei were stained with 500× diluted DAPI (in DEPC-H_2_O) for 5 min, washed three times with PBS (5 min each), treated with anti-quencher, coverslipped, sealed with nail polish, and observed using laser confocal microscopy.

### 4.3. Construction of VIGS Vectors for Pear FWL Genes

We utilized TRV-based vectors, specifically pTRV1 and pTRV2. The pTRV2 vector was linearized through digestion with EcoRI and BamHI enzymes. We designed specific primers targeting the full-length sequences of FWL1 and FWL5 for virus-induced gene silencing (VIGS) (Table A2). Amplification was conducted using cDNA synthesized from the flesh tissue of the Korla fragrant pear as the template. The primers incorporated homologous sequences complementary to the linearized vector. Both the linearized vector and the resulting PCR products underwent purification using a DNA Gel Extraction Kit (Vazyme Biotech Co., Ltd., Nanjing, China). Subsequently, these components were ligated at 50 °C for 30 min using a ClonExpress Ultra One Step Cloning Kit V3 (Vazyme Biotech Co., Ltd., Nanjing, China), resulting in the formation of circular recombinant plasmids, TRV2-FWL1 and TRV2-FWL5.

### 4.4. VIGS of FWL Genes

The successfully transformed GV3101 strain containing pTRV2-FWL1/5 vectors was cultured in YEP medium, supplemented with 10 mM MES, 20 mM acetosyringone, 25 µg/mL rifampicin, and 50 µg/mL kanamycin, and incubated at 28 °C for 24 h. Following this, the Agrobacterium cells were harvested via centrifugation and resuspended in an infiltration buffer (OD600 = 1.2) containing 200 mM acetosyringone, 10 mM MgCl_2_, and 10 mM MES (pH 5.6). Cultures containing pTRV1 with pTRV2 (control) and pTRV1 with pTRV2-FWL1/5 were mixed at a 1:1 (*v*/*v*) ratio and allowed to sit in the dark for 4 to 6 h prior to the inoculation of young Korla fragrant pear fruits (10 DAFB). Then, 10 days post-infection, pulp tissues were collected for RT-PCR analysis. Thirty samples from each treatment were fixed in an FAA solution, subjected to a graded ethanol dehydration series, and subsequently embedded in paraffin wax. Thin sections (10–12 µm) were prepared using a rotary microtome, flattened onto microscope slides, dewaxed, and stained with safranin and fast green. All histological preparations were examined and photographed using a Nikon Eclipse 80i microscope (Nikon Corporation, Tokyo, Japan) at 4×/0.10 magnification. To assess the average cell volume, the width and length of cells per square millimeter were measured from microscopic images corresponding to the flesh. Cell dimensions were determined through crosswise and lengthwise analyses of 6 randomly selected areas within the fruit mesocarp images, explicitly excluding the epidermis, hypodermis, and vascular bundles. The cell volume was calculated using the following formula: V = 4/3πd^3^, where d represents half the average of the measured cell width and length. The average cell count was subsequently derived from the ratio of fruit volume to cell volume. Statistical analyses were performed using one-way ANOVA with Tukey’s test for post hoc comparisons.

### 4.5. Library Construction and Protein Screening

Young fruit pulp tissues of Duli pear, Korla fragrant pear, and Yali pear were meticulously ground into a fine powder using liquid nitrogen. Total RNA was extracted employing the RNAprep Pure Polysaccharide Polyphenol Plant Total RNA Extraction Kit (Tiangen Biotech (Beijing) Co., Ltd., Beijing, China). Subsequently, the extracted RNA was reverse-transcribed into complementary DNA (cDNA) utilizing the FastQuant cDNA First-Strand Synthesis Kit (Tiangen Biotech (Beijing) Co., Ltd., Beijing, China). Following this, the PCR products underwent purification using a QIAquick PCR Purification Kit (Shang Hai Haoran Biological Technology Co., Ltd., Shanghai, China) after homogenization. For further details on the library construction and protein screening methodologies, please refer to Sai et al. [27].

### 4.6. Construction of Decoy Expression Vectors and Self-Activation Assay

**Figure A1:**
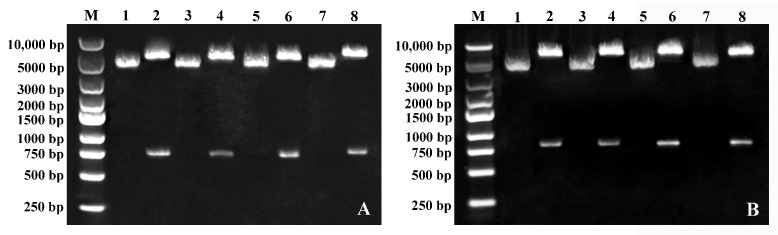
Decoy vector double digestion. M is marker, A1–2 are T-pBT3-N-FWL1, A3–4 are pBT3-C-FWL1, A5–6 are pBT3-SUC-FWL1, A7–8 are pBT3-STE-FWL1; B1–2 are T-pBT3-N-FWL5, B3–4 are pBT3-C-FWL5, B5–6 are pBT3- SUC-FWL5, and B7–8 are T-pBT3-STE-FWL5, where 1, 3, 5, and 7 of (**A**,**B**) are circular recombinant plasmids, and 2, 4, 6, and 8 are double-enzymatically cleaved linear bands.

Building upon the pear fruit size-related genes FWL1 and FWL5, which were previously cloned by our research group [15,26], we designed full-length design primers specific to the FWL1 and FWL5 genes tailored to four vectors, namely, pBT3-N, pBT3-C, pBT3-SUC, and pBT3-STE (Table A2). The PCR reaction was conducted using cDNA as the template, with subsequent purification and recovery of the PCR products. The pBT3-N/C/SUC/STE vectors were enzymatically digested (Figure A1), allowing for ligation of the PCR product with the linearized vectors via DNA ligase. This mixture was transformed into Top10 competent cells, which were plated on the associated resistance plate. The bacterial solution was subjected to PCR screening after an overnight incubation at 37 °C with gentle shaking. Positive colonies from each plate were subsequently picked and cultivated in the respective LB liquid media supplemented with appropriate antibiotics. The resulting cultures were incubated at 37 °C and agitated at 220 rpm for 12 h. Plasmid extraction and sequencing of positive clones were performed using the corresponding primers. The construction of the Ole-e-1 vector was executed in a parallel manner, adhering to the same protocol (Table A2).

Using the Saccharomyces cerevisiae Receptor Preparation and Transformation Kit (Item No. PT1183, Puri Biotechnology Co., Ltd., Yangling, China), the NMY51 yeast strain underwent receptor preparation. A mixture of carrier DNA, pTSU2-APP, and pNubG-Fe65 was incorporated into the NMY51 yeast strain alongside a PEG/LiAc transformation solution. This preparation was plated on DDO (SD/-Leu/-Trp) and QDO/X (SD/-Leu/-Trp/-His/-Ade/X-a-gal) media specifically designed to host pTSU2-APP and pNubG-Fe65 constructs. Following this, the constructed pBT3-N/C/SUC/STE-FWLs vectors were combined with pOst1-NubI and pPR3-N. The addition of carrier DNA (10 μg/mL) resulted in the formation of 8 distinct groups (Table A3). Each group was treated with 50 μL of NMY51 yeast and PEG/LiAc transformation solution, then plated on DDO (SD/-Leu/-Trp) and QDO/X (SD/-Leu/-Trp/-His/-Ade/X-a-Gal). For further assays, 100 μL from each group (1 through 8) was also plated on SD/-Leu agar, which was then subjected to functional and self-activation assessments. For the next step, an EP tube containing 500 μL of 0.9% NaCl solution was prepared. A robust colony from the transformed NMY51 (pBT3-N-FWL&pPR3-N) plate was selected and suspended in the NaCl solution. Serial dilutions to OD values of 0.1 and 0.01 were performed, followed by spotting of the bacterial solution onto DDO and varying concentrations of 3-AT on QDO/X plates (10 mM, 20 mM, 30 mM, 40 mM, and 50 mM). The plates were incubated at 30 °C for 3 days to monitor growth characteristics.

### 4.7. Fluorescence Quantification

The expression of genes associated with *FWL1* and *FWL5*, along with their interactions with candidate proteins, was investigated using quantitative reverse transcription polymerase chain reaction (qRT-PCR). *UBI* was chosen as the internal reference gene [49]. Primers for qRT-PCR were designed with DNAMAN software (Version 10) (Table A2). Total RNA was extracted from pulp tissue samples collected during the critical cell division phase in the fruit of Duli pear, Korla fragrant pear, and Yali pear. The extraction was performed using the RNAprep Pure Polysaccharide Polyphenol Plant Total RNA Extraction Kit (Tiangen Biotech (Beijing) Co., Ltd., Beijing, China). After measuring the RNA concentration with NanoDrop One/OneC ND-1000, the concentration was standardized across samples. Subsequently, the RNA was reverse transcribed into cDNA utilizing the FastQuant cDNA First Strand Synthesis Kit (Tiangen Biotech (Beijing) Co., Ltd., Beijing, China), and was diluted 10-fold for subsequent qRT-PCR analysis. The qRT-PCR was conducted with BlasTaq 2×qPCR MasterMix (Applied Biological Materials Inc., Richmond, BC, Canada), following this reaction setup: 0.5 μL of each upstream and downstream primer, 1 μL of template, and 10 μL of BlasTag 2×qPCR MM1, with the final volume adjusted to 20 μL using Nuclease-free H_2_O. The qRT-PCR program included an initial pre-denaturation at 95 °C for 3 min, followed by denaturation at 95 °C for 15 s, annealing at 57 °C for 15 s, and extension at 60 °C for 20 s, with a total of 35 cycles. Fluorescent signals were collected at the 3rd step of each cycle. The mixed sample was divided into three portions, with three technical replicates set up. Data analysis was performed using TBtools (Version 1.115) software (adopting one-way analysis of variance (ANOVA) combined with Tukey’s post hoc test, and the significance threshold was set at *p* < 0.05), followed by a statistical significance analysis and result visualization.

### 4.8. Yeast Two-Hybrid Validation

Candidate proteins were cloned into bait vectors, specifically the pBT3 and pPR3 vectors for FWL and the identified intercalating proteins, respectively. These constructs were co-transformed into yeast strains, which were subsequently plated on SD/-Leu/-Trp media. The plates were inverted and incubated at 30 °C for 5 days to facilitate growth observation. A well-isolated colony from each plate was selected and transferred to an EP tube containing 500 μL of 0.9% NaCl solution. After thorough mixing, the solution was diluted to achieve an OD value of 0.1. A volume of 2.5 μL of this suspension was spotted onto various selective media: SD/-Trp/-Leu, SD/-Trp/-Leu/-His/X-a-gal/50mM3-AT, and repeated for SD/-Trp/-Leu/-His/-Ade/X-a-AT plates. These plates were then incubated at 30 °C for 3 days to assess the growth status of the yeast.

### 4.9. Bimolecular Fluorescence Complementation (BiFC)

Homologous recombination primers, designed without stop codons in the open reading frames (ORFs) of the FWL1, FWL5, and Ole-e-1 genes, were synthesized (Table A2). Using these primers, the bimolecular fluorescence complementation (BiFC) vectors pEarleyGate201-YN and pEarleyGate202-YC were successfully constructed, and the recombinant plasmids, namely pEarleyGate201-YN-FWL1, pEarleyGate201-YN-FWL5, and pEarleyGate202-YC-Ole-e-1, were finally obtained. The resulting fusion expression vectors were transformed into the Agrobacterium tumefaciens strain GV3101. For transient expression in tobacco leaves (*Nicotiana benthamiana*), the combinations FWL1-YN&YC, FWL5-YN&YC, Ole-e-1-YN&YC, and YN&YC were used as negative controls. After the transformation was completed, the tobacco leaves were observed using a laser confocal microscope, and images were captured for subsequent analysis. A laser confocal microscope was used for YFP (Yellow Fluorescent Protein) imaging, with an excitation/emission wavelength of 510 nm, under a 20× oil-immersion objective lens.

### 4.10. Candidate Gene Biosignature Analysis

The sequenced samples were rigorously validated through BLAST comparison on the NCBI website (https://blast.ncbi.nlm.nih.gov/, accessed on 10 March 2023). Sequence analysis and amino acid translation were conducted utilizing DNAMAN v6, complemented by online software tools such as Prot Param (https://web.expasy.org/protparam, accessed on 10 March 2023), which facilitated the evaluation of the relative molecular weight, isoelectric point, instability coefficient, and hydrophilic mean for the Ole_e_1 protein. The analysis of the protein’s transmembrane regions was performed using TMHMM-2.0 (https://services.healthtech.dtu.dk/services/TMHMM-2.0/, accessed on 11 March 2023), while the signal peptide characteristics were investigated with SignaIP 4.1 (https://www.cbs.dtu.dk/services/SignalP-4.1/, accessed on 12 March 2023) for predicting protein signal peptides. Additionally, protein hydrophilicity assessments were conducted using ExPASy-ProtScale (https://web.expasy.org/protscale/, accessed on 12 March 2023) and analyzed for phosphorylation site prediction via NetPhos-3.1 (https://services.healthtech.dtu.dk/services/NetPhos-3.1/, accessed on 13 March 2023). Structural domain analysis of the protein was facilitated through CCD (http://www.ncbi.nlm.nih.gov/cdd, accessed on 14 March 2023) available on the NCBI website. Furthermore, the subcellular localization of the Ole_e_1 protein was predicted using the online tool Cell-PLoc 2.0 (http://www.csbio.sjtu.edu.cn/bioinf/Cell-PLoc-2/, accessed on 15 March 2023). The Ole-e-1 protein sequence was also cross-referenced through the UniProt database (https://www.uniprot.org/, accessed on 16 March 2023), allowing for a data export of analogous genes. Subsequently, TBtools software was employed to construct an evolutionary tree for these genes, from which 5 proteins were randomly selected from disparate subfamilies for comprehensive gene structure and motif analysis.

## 5. Conclusions

*FWL1* and *FWL5* exhibit expression in the core and the flesh adjacent to the core of the pear. Notably, hybridization signals obtained from small fruits surpass those from large fruits. Upon silencing of the *FWL5* gene, the number of cells increases significantly, accompanied by a corresponding enlargement of the fruit. *FWL5* demonstrates a strong correlation with the interacting proteins previously identified for *FWL1*, including a gene designated as *Ole-e-1*, which displays a positive association with pear fruit size throughout the process of fruit cell division. Validation through yeast two-hybrid and BiFC methodologies has substantiated the interaction of Ole-e-1 with both FWL1 and FWL5. These findings highlight the potential roles of *FWL1*, *FWL5*, and *Ole-e-1* in pear fruit cell division and the regulation of fruit size, providing a theoretical basis for targeted breeding to increase pear fruit size.

## Figures and Tables

**Table 1:** Determination of fruit size, cell size, and cell number following FWL gene silencing.

Treatment	Fruit Weight (g)	Fruit Volume (mm^3^)	Cell Volume (×10^−6^ mm^3^)	Cell Numbers (×10^4^)
pTRV2-FWL1	1.13 ± 0.04 b	596.98 ± 30.54 b	20.65 ± 0.62 a	2890.94 ± 75.78 b
pTRV2-FWL5	1.58 ± 0.56 a	663.33 ± 34.65 a	18.94 ± 0.39 b	3502.27 ± 83.44 a
pTRV2	1.18 ± 0.09 b	608.82 ± 31.70 b	20.43 ± 0.60 a	2980.03 ± 73.45 b
nature	1.12 ± 0.06 b	589.65 ± 28.36 b	20.75 ± 0.53 a	2841.69 ± 69.78 b

Note: Different lowercase letters (a, b) indicate significant differences at the *p* < 0.05.

**Table 2:** List of proteins that interact with FWL5 protein (top 10).

Number	Accession	Marginal Notes	Frequency
1	XM_009352003.2	metallothionein-like protein type 2	7
2	XM_009367135.2	uncharacterized	6
3	XM_009379993.2	collagen and calcium-binding EGF domain-containing protein 1-like	5
4	XM_009379993.3	branchpoint-bridging protein-like	5
5	BX640903.1	Homo sapiens mRNA	3
6	GQ906589.1	metallothionein-like protein (Met1)	3
7	NM_001302326.1	auxin-repressed 12.5 kDa protein-like (ARP)	3
8	XM_008343913.3	small ubiquitin-related modifier 1-like	3
9	XM_009337215.2	V-type proton ATPase 16 kDa proteolipid subunit-like	3
10	XM_009340070.2	aquaporin TIP1-3-like	3

## Data Availability

All data that support the findings of this study are available from the corresponding author upon reasonable request.

## Appendix A

**Table A1:** Sequence information for FISH in situ hybridization probe preparation.

Gene Name	Sequence (5′-3′)
*FWL1*	CATTTTGGAGCGGTAGAAGCATGAGTAGCAGCATGGACAAGCTGT
*FWL5*	CAACGACTGCCTAAACAGACCTGTGTAGATTGCACACATCCACCA

**Table A2:** Primer sequences used in this study.

Primers	Primers Sequence (5′-3′)
*FWL1*-F	CGGAATTCGACAGGTATCCCGGTCAGCT
*FWL1*-R	CGGGATCCCCAAGGGATGAGAGCACGA
*FWL5*-F	CGGAATTCGAGAGTTGCTGGACTGGACT
*FWL5*-R	CGGGATCCGCAATTCCTCCTTCAACGCA
FWL1-F (pBT3-N)	GAATTCCTGCAGGGCCATTACGGCCATGTACTCGTCACACCCAAGGG
FWL1-R (pBT3-N)	TACTTACCATGGGGCCGAGGCGGCTTTATCTCGACTCATGCCTTCCTC
FWL1-F (pBT3-C)	CACTAATCTAGACGGCCATTACGGCCATGTACTCGTCACACCCAAGGG
FWL1-R (pBT3-C)	ATGGAGGCCTTTGGCCGAGGCGGCTTTATCTCGACTCATGCCTTCCTC
FWL1-F (pBT3-SUC)	AATATCTGCAATGGCCATTACGGCCATGTACTCGTCACACCCAAGGG
FWL1-R (pBT3-SUC)	ATTCCTGCAGATGGCCGAGGCGGCTTTATCTCGACTCATGCCTTCCTC
FWL1-F (pBT3-STE)	TATTTTATGTAATGGCCATTACGGCCATGTACTCGTCACACCCAAGGG
FWL1-R (pBT3-STE)	ATTCCTGCAGATGGCCGAGGCGGCTTTATCTCGACTCATGCCTTCCTC
FWL5-F (pBT3-N)	GAATTCCTGCAGGGCCATTACGGCCATGGCAGAAGGGAATCCGCA
FWL5-R (pBT3-N)	TACTTACCATGGGGCCGAGGCGGCCACAGGCTGCAGAGCCAG
FWL5-F (pBT3-C)	CACTAATCTAGACGGCCATTACGGCCATGGCAGAAGGGAATCCGCA
FWL5-R (pBT3-C)	ATGGAGGCCTTTGGCCGAGGCGGCCACAGGCTGCAGAGCCAG
FWL5-F (pBT3-SUC)	AATATCTGCAATGGCCATTACGGCCATGGCAGAAGGGAATCCGCA
FWL5-R (pBT3-SUC)	ATTCCTGCAGATGGCCGAGGCGGCCACAGGCTGCAGAGCCAG
FWL5-F (pBT3-STE)	TATTTTATGTAATGGCCATTACGGCCATGGCAGAAGGGAATCCGCA
FWL5-R (pBT3-STE)	ATTCCTGCAGATGGCCGAGGCGGCCACAGGCTGCAGAGCCAG
pPR3-N-Ole-e-1-F	TGTTCCAGATTACGCTGGATCCATGTCTTGGTTTCTTACAGTTATCTTTCTGG
pPR3-N-Ole-e-1-R	ACTAATTACATGACTCGAGGTCGACTCATGGTGCTGAACCCGAT
*MT*-F	GTGGTAAATGTGGTTGCGGGTC
*MT*-R	CCGCACTTGCATCCGTTCTC
*Ole-e-1*-F	GCCTTCTGCAGTAATTGTTGGC
*Ole-e-1*-R	GTCCCATCTTTGCATTCCACACC
*UBI*-F	GCTCGCAGTGCTCCAGTTCTAC
*UBI*-R	AACATAGGTCAACCCGCACTT
pEarleyGate201-YN-FWL1-F	ACAAGTTTGTACAAAAAAATGTACTCGTCACACCCAAGG
pEarleyGate201-YN-FWL1-R	CACCACTTTGTACAAGAATTTATCTCGACTCATGCCTTCC
pEarleyGate201-YN-FWL5-F	ACAAGTTTGTACAAAAAAATGGCAGAAGGGAATCCG
pEarleyGate201-YN-FWL5-R	CACCACTTTGTACAAGAACACAGGCTGCAGAGCCAGCT
pEarleyGate202-YC-Ole-e-1-F	ACAAGTTTGTACAAAAAAATGTCTTGGTTTCTTACAGTTATCTTTCTGG
pEarleyGate202-YC-Ole-e-1-R	CACCACTTTGTACAAGAATGGTGCTGAACCCGATGGT

**Table A3:** Yeast two-hybrid plasmid combinations.

Combination	Plasmid Combination	Function
1	pBT3-N-FWL5&pOst1-NubI	Functional testing
2	pBT3-C-FWL5&pOst1-NubI	Functional testing
3	pBT3-SUC-FWL5&pOst1-NubI	Functional testing
4	pBT3-STE-FWL5&pOst1-NubI	Functional testing
5	pBT3-N-FWL5&pPR3-N	Self-activation detection
6	pBT3-C-FWL5&pPR3-N	Self-activation detection
7	pBT3-SUC-FWL5&pPR3-N	Self-activation detection
8	pBT3-STE-FWL5&pPR3-N	Self-activation detection

Note: Add 1000 ng of each plasmid in each group.
